# Generative AI and criminology: A threat or a promise? Exploring the potential and pitfalls in the identification of Techniques of Neutralization (ToN)

**DOI:** 10.1371/journal.pone.0319793

**Published:** 2025-04-04

**Authors:** Federico Pacchioni, Emma Flutti, Palmina Caruso, Lorenzo Fregna, Francesco Attanasio, Carolina Passani, Cristina Colombo, Guido Travaini

**Affiliations:** 1 Vita-Salute San Raffaele University, Milan, Italy; 2 Department of Human Neuroscience, Sapienza University of Rome, Rome, Italy; 3 University of Milan, Milan, Italy; 4 Department of Clinical Neuroscience, Mood Disorders Unit, IRCCS San Raffaele Scientific Institute, Milan, Italy; University of KwaZulu-Natal College of Health Sciences, SOUTH AFRICA

## Abstract

Generative artificial intelligence (AI) such as GPT-4 refers to systems able to understand and generate new coherent and relevant text by learning from existing data sets. The great opportunities that GPT-4 offers are accompanied by great risks. Indeed, the ease of access and use of such a tool also makes it the platform of choice for malicious users. The purpose of this work is to test the machine’s capabilities in identifying and reframing so-called Techniques of Neutralization (ToN), rationalizations employed by offenders to justify their deviant behavior. The identification of such theoretical cornerstone of criminology in interviews with offenders is crucial for criminologists, as it provides relevant information on criminodynamics, risk assessment and possible intervention strategies. Our outcomes show a high level of the machine’s accuracy in general ToN recognition of Published and Crafted ToN sentences in both Test 1 (precision of 0.82 and recall of 0.75 for “Denial of Injury” in Crafted ToN, precision of 0.93 and recall of 1 for “Absence of ToN” in Published ToN) and Test 2 (precision of 1.00 and recall of 0.83 for “Denial of Injury” in Crafted ToN, precision of 1.00 and recall of 1.00 for “Absence of ToN” in both ToN categories). Regarding the reformulation of sentences to remove ToN (Test 3), the model demonstrates high success rates for most ToN categories and high credibility of the reformulated sentences, indicating its ability to maintain the integrity of the sentences while removing the ToN. Our work analyses the application of the machine with respect to a previously untested construct, with the aim of observing the potential and, above all, the possible pitfalls behind the use of AI models in a hitherto little-explored context such as criminology.

## Introduction

Amidst a heated debate regarding the definition of Generative Artificial Intelligence (GAI), a recent systematic mapping has allowed its characterization as “the production of previously unseen synthetic content, in any form and to support any task, through generative modeling” [1]. The focus is on the process of generating new content from existing resources rather than solely on the generated content itself. Leveraging tools such as ChatGPT, Dall-E, or Midjourney has garnered significant attention for their ability to create new content resembling human-like quality, learning from existing datasets [1–3]. These tools have demonstrated their potential across various applications, including natural language processing and text analysis [2–4]. Against this backdrop, Chat GPT was launched, opening a new frontier in the development of artificial intelligence, proving to be an innovative and highly successful product, capable of answering users’ questions [5]. Chat GPT is an Artificial Intelligence (AI) model based on the family of Natural Language Processing (NLP) models, developed by OpenAI agency [6]. A study by UBS, based on Similarweb data, called Chat GPT “the fastest growing software” [7]. The latest available version of Chat GPT is ChatGPT-4 which is trained on a great repertory of linguistic data to understand context and generate coherent and contextually relevant text, making them valuable tools for various applications, including natural language processing and text analysis [6,8]. Following the advent of a new AI tool, academics have debated its impact, particularly on job displacement [9–11]. The Trade and Technology Council (TCC) notes AI’s potential for job creation and productivity increases, but also its disruptive potential [12]. The role of AI, specifically Chat GPT, in job markets is complex and needs more study. Given the remarkable capabilities of generative artificial intelligence models such as Chat GPT, we were intrigued not only by the potential of such models, but also wondered about the possible pitfalls of their application for unethical, improper and unlawful purposes. It was thus decided to import the model into our world of interest, fascinated by the possible outcomes of the encounter between Criminology and Chat GPT. The field of criminology was chosen as a testing ground as it is a branch that more than others lends itself to verifying both the potential benefits and the negative implications of AI models [13]. We decided to consider a theoretical cornerstone of criminology, which is Techniques of Neutralization (ToN). These have been proposed by Sykes and Matza [14] and defined as cognitive strategies that individuals use to rationalize and justify their behavior, particularly when such actions are illicit. The original techniques were five: Denial of Responsibility (“It’s not my fault”), Denial of Injury (“No one got hurt”), Denial of the Victim (“They deserved it”), Condemnation of the Condemners (“The law enforcers are corrupt”) and Appeal to Higher Loyalties (“I did it for my family”). These techniques are valuable in understanding how an individual can lessen cognitive dissonance - conflict arising from the gap between actions and moral beliefs - which can subsequently play a pivotal role in formulating the justifications that underpin criminal behavior [15–17]. In effect, the perpetrator enacts a rationalization process that allows them to act in a deviant manner, “neutralizing” any conflict with societal moral norms. These techniques often result in the dismissal of personal accountability and the refutation of the illicit nature of their actions.

Detecting the use of ToN in offender discourses is integral to criminologists. It offers insights into the motivations and justifications behind criminal behaviors, helping shape effective risk assessments and design intervention strategies [18–20]. This is crucial for evaluating the offender’s potential for reform and understanding what kind of rehabilitative approach might be most effective.

Given their intricate and multifaceted nature, these mechanisms make an excellent case for testing the capabilities of generative AI models.

This study holds important implications across multiple domains. From a social perspective, it enhances understanding of the mechanisms underlying justification in criminal behavior, offering valuable insights that may inform the development of prevention strategies and public awareness initiatives. Academically, the research bridges a critical gap at the intersection of criminology and AI, establishing a foundation for future investigations into the application of advanced models such as ChatGPT in social science contexts. Ethically, the findings emphasize the necessity of addressing and mitigating potential risks associated with the misuse of AI in sensitive areas, reinforcing the importance of promoting responsible and ethical applications of this technology.

## Materials and methods

This study was designed to evaluate the capability of ChatGPT in identifying Neutralization Techniques (ToN) and reformulating sentences containing them. The experimental procedure was divided into three distinct sample groups and three test phases.

### Samples

The sample consisted of 200 sentences categorized into three sets. The characteristics of each subset, including quantity, description, creation method, and confirmation method, are summarized in Table 1.

**Table 1:** Summary of Sentence Samples Characteristics. ToN: Techniques of Neutralization.

Sample	Quantity	Description	Creation Method	Labeling/Confirmation Method
**Published ToN Sentences** **(Sample 1)**	50 sentences	Sentences extracted from various scholarly articles and documents, exhibiting ToN	Extracted from scholarly articles and documents	Labelled based on original document descriptions; Identifying the presence and type of ToN
**Crafted ToN Sentences** **(Sample 2)**	50 sentences	New sentences crafted to include ToN	Created by expert criminologists	Evaluated and labelled by another expert criminologist in a blind review
**Neutral Control Sentences** **(Sample 3)**	100 sentences	Neutral control sentences without ToN	Created by expert criminologists	Evaluated and confirmed by a second criminologist to ensure absence of ToN

The complete texts of the 200 sentences are provided in the Supplementary Materials.

### Procedure

Three tests were carried out as follows:

- Test 1 (“Published ToN Identification Test”): ChatGPT was given a randomized sequence of 50 sentences from Sample 1 (“Published ToN Sentences”) and 50 randomly chosen sentences from Sample 3 (“Neutral Control Sentences”). The model was tasked with evaluating each sentence to determine whether or not it contained a ToN. In cases where no ToN were present, the model was expected to indicate this. If a ToN was present, the model was required to identify the specific category of ToN.
- Test 2 (“Crafted ToN Identification Test”): ChatGPT received a randomized sequence of 50 sentences from Sample 2 (“Crafted ToN Sentences”) and 50 randomly chosen sentences from Sample 3, with the same instructions as in Test 1.
- Test 3 (“ToN Reformulation Test”): For every sentence identified as containing a ToN in Tests 1 and 2, ChatGPT was tasked with formulating an alternative sentence that didn’t contain a ToN. The model was instructed to strive to alter the content of the sentence as minimally as possible while maintaining the sentence’s credibility. An exemplary demonstration of this task can be seen with the sentence “I did It (referring to the offence) because I was concerned that my family’s honor was taken. […] They took my honor,” which contained an ‘Appeal to higher loyalties’ ToN. A correctly reformulated sentence is: “I felt as though my family’s honor was compromised. It seemed to me that my honor had been taken away. Nonetheless, I understand that honor and personal actions are distinct, and my actions remain my own responsibility.”

The model’s proficiency in reformulating sentences was assessed based on two main criteria: the absence of Techniques of Neutralization (ToN) and the credibility of the reformulated sentences. The evaluation of the reworded sentences for ToN absence was conducted independently by two expert criminologists, FE and CP. In cases of disagreement, a third opinion was sought from another criminologist, TG. The credibility of the reformulated sentences was evaluated by three criminology experts (FE, CP, and TG) who each assigned a score ranging from 0 to 5, based on several criteria. These criteria encompassed the plausibility of the sentence in a real-world context, the preservation of the original intent and meaning, as well as the effective removal of Techniques of Neutralization (ToN). The final reported score represents the average of the scores assigned by the three independent criminologists. This comprehensive evaluation approach provided a nuanced understanding of the model’s performance in reformulating sentences, ensuring both the removal of ToN and the maintenance of sentence credibility. No prompt engineering techniques were applied to the instructions.

**Fig 1:**
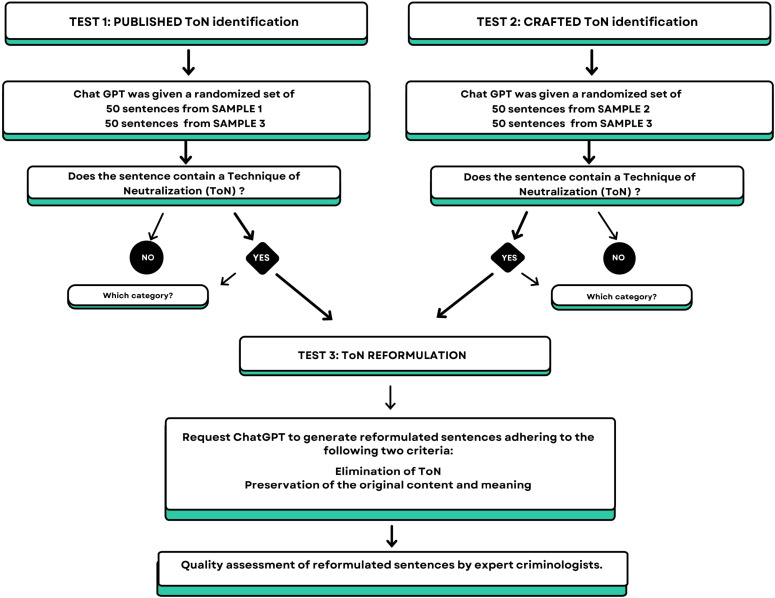
The figure schematically represents the experimental design. Sample 1: 50 sentences containing ToN taken from published material; Sample 2: 50 sentences containing ToN crafted by criminologists; Sample 3: 100 neutral sentences without ToN.

To prevent ChatGPT from learning the pattern of the sentences being tested, each sentence was presented in a new chat session with the chat history disabled. The design of the experiment is shown in Fig 1.

### Analysis

The performance of ChatGPT was evaluated using precision, recall, and F1 score metrics.

In Test 1 and Test 2, the model’s performance was assessed based on its ability to correctly identify the presence or absence of ToN in sentences from the Published ToN and Neutral Control Sentences samples (Test 1), and the Crafted ToN and Neutral Control Sentences samples (Test 2). Furthermore, the model’s ability to correctly identify the specific category of ToN, as originally defined by Sykes and Matza [13], was also evaluated. The precision, recall, and F1 scores were calculated by comparing the model’s predictions with the original labels assigned by the expert criminologists.

In Test 3, the model’s ability to reformulate sentences containing ToN was assessed. The quality of the reformulated sentences was evaluated based on whether the model successfully removed the ToN without significantly altering the original content of the sentence.

Discrepancies between the model’s predictions and the original labels were resolved through a review process, where the sentences were re-evaluated by the expert criminologists.

The analyses were conducted in May 2023 using ChatGPT (specifically, the GPT-4 model), Excel, and R. For Tests 1 and 2, the scores assigned by the criminologists were subsequently used to create a confusion matrix. In Test 3, the scores were analyzed descriptively to evaluate the model’s ability to reformulate sentences containing ToN. The results were tabulated and visualized for a more comprehensive understanding of the model’s performance. Examples of prompts and possible responses are available in the supplementary material.

## Results

The results are divided into three parts:

1. **General ToN Recognition** (Tests 1 and 2), which gauged the model’s capability in detecting the presence or absence of ToN and correctly identifying the specific ToN category.
2. **Specific ToN Category Recognition** (Tests 1 and 2), evaluating the model’s ability to detect the ToN and accurately pinpoint the specific ToN category present.
3. **ToN Reformulation Analysis** (Test 3), testing the model’s ability to reformulate sentences containing ToN into sentences devoid of ToN, while preserving the original sentence content.

In the General ToN Recognition Test 1, the model correctly identified 46 out of 50 ToN sentences and made no false positives. However, it failed to identify 4 ToN sentences. In Test 2, the model perfectly identified all ToN and Non-ToN sentences, indicating a high level of accuracy in identifying crafted ToN sentences (Table 2).

**Table 2:** General ToN Recognition (Tests 1 and 2).

Test 1: “Published ToN Identification Test”		
	Predicted ToN	Predicted Non-ToN
**Actual ToN**	46 (TP)	4 (FN)
**Actual Non-ToN**	0 (FP)	50 (TN)
**Test 2: “Crafted ToN Identification Test”**		
	**Predicted ToN**	**Predicted Non-ToN**
**Actual ToN**	50 (TP)	0 (FN)
**Actual Non-ToN**	0 (FP)	50 (TN)

Table 2. Confusion matrices for Test 1 (Published ToN Identification Test) and Test 2 (Crafted ToN Identification Test), showing the number of True Positives (TP), False Negatives (FN), False Positives (FP), and True Negatives (TN).

The metrics table (Table 3) provides a quantitative evaluation of the model’s performance in the General ToN Recognition tests. In both tests, the model demonstrated perfect precision, indicating high accuracy in identifying sentences containing ToN. The recall was slightly lower in the Published ToN Identification Test, suggesting a few instances of missed ToN sentences. However, in the Crafted ToN Identification Test, the model achieved perfect recall, indicating an excellent ability to identify all crafted ToN sentences. The F1 score, a measure that balances precision and recall, was slightly lower in the Published ToN Identification Test due to the lower recall but reached perfection in the Crafted ToN Identification Test.

**Table 3:** The table presents the precision, recall, specificity, and F1 score for Test 1 and Test 2.

Metrics	Published ToN	Crafted ToN
**Precision**	1	1
**Recall**	0.92	1
**Specificity**	1	1
**F1 Score**	0.96	1

### Specific ToN category recognition (Tests 1 and 2)

Table 4, a confusion matrix for the Specific ToN Category Recognition in Test 1, indicates that the model’s success in identifying specific ToN categories varied. It correctly recognized 9 of 12 “Denial of Injury” sentences, 6 of 14 “Denial of Victim” sentences, 9 of 13 “Denial of Responsibility” sentences, none of the 4 “Condemn of the Condemners” sentences, 6 of 7 “Appeal to Higher Loyalties” sentences, and accurately identified all 50 sentences that had an absence of ToN.

**Table 4:** Confusion matrix for the Specific ToN Category Recognition in the Published ToN Identification Test (Test 1).

Predicted Actual	Denial of Injury	Denial of Victim	Denial of Responsibility	Condemn of the condemners	Appeal to Higher Loyalties	Absence of ToN
**Denial of Injury (12)**	9	0	0	0	3	0
**Denial of Victim (14)**	2	6	4	0	0	2
**Denial of Responsibility (13)**	0	0	9	1	1	2
**Condemn of the condemners (4)**	0	2	1	0	1	0
**Appeal to Higher Loyalties (7)**	0	0	1	0	6	0
**Absence of ToN (50)**	0	0	0	0	0	50

The table shows the number of sentences correctly and incorrectly classified into each category of ToN. The number in represents the total number of sentences presented for each class.

The metrics table (Table 5) provides a quantitative evaluation of the model’s performance in recognizing specific categories of ToN in Test 1. The model demonstrated high precision and recall for “Denial of Injury”, “Absence of ToN” categories, resulting in high F1 scores. However, the model struggled with the “Denial of Victim”, “Denial of Responsibility”, “Appeal to Higher Loyalties”, and “Condemn of the Condemners” categories, as indicated by lower precision, recall, and F1 scores. Particularly, the model was able to identify some but not all sentences in the “Condemn of the Condemners” category, resulting in an undefined F1 score. These results indicate that while the model was highly accurate in some categories, it had difficulties in others.

**Table 5:** Precision, Recall, and F1 Score for each category of ToN and Absence of ToN in the Published ToN Identification Test (Test 1).

Class	Precision	Recall	F1 Score
**Denial of Injury**	0,82	0,75	0,78
**Denial of Victim**	0,75	0,43	0,54
**Denial of Responsibility**	0,6	0,69	0,64
**Condemn of the Condemners**	0	0	Undefined
**Appeal to Higher Loyalties**	0,54	0,86	0,66
**Absence of ToN**	0,93	1	0,96

The confusion matrix for the Specific ToN Category Recognition in Test 2 reveals varied performance levels (Table 6). The model accurately identified 10 of 12 “Denial of Injury” sentences, 11 of 12 “Denial of Victim” sentences, all 10 “Denial of Responsibility” sentences, 2 of 8 “Condemn of the Condemners” sentences, 7 of 7 “Appeal to Higher Loyalties” sentences, and every sentence without ToN.

**Table 6:** Confusion matrix for the Specific ToN Category Recognition in the Crafted ToN Identification Test (Test 2).

Predicted Actual	Denial of Injury	Denial of Victim	Denial of Responsibility	Condemn of the Condemners	Appeal to Higher Loyalties	Absence of ToN
**Denial of Injury (12)**	10	2	0	0	0	0
**Denial of Victim (12)**	0	11	0	0	1	0
**Denial of Responsibility (10)**	0	0	10	0	0	0
**Condemn of the Condemners (8)**	0	0	4	2	2	0
**Appeal to Higher Loyalties (7)**	0	0	0	0	7	0
**Absence of ToN (50)**	0	0	0	0	0	50

The table shows the number of sentences correctly and incorrectly classified into each category of ToN and Absence of ToN. The number in represents the total number of sentences presented for each class.

The metrics table (Table 7) provides a quantitative evaluation of the model’s performance in recognizing specific categories of ToN in Test 2. The model demonstrated high precision and recall for “Denial of Injury”, “Denial of Responsibility”, “Appeal to Higher Loyalties”, and “Absence of ToN” categories, resulting in high F1 scores. However, the model struggled with the “Condemn of the Condemners” category, as indicated by lower precision, recall, and F1 scores. Particularly, the model was unable to correctly identify a significant number of sentences in the “Condemn of the Condemners” category, resulting in a 0.40 F1 score. These results indicate that while the model was highly accurate in some categories, it had difficulties in others.

**Table 7:** Precision, Recall, and F1 Score for each category of Techniques of Neutralization (ToN) and Absence of ToN in the Crafted ToN Identification Test (Test 2).

Class	Precision	Recall	F1 Score
**Denial of Injury**	1,00	0,83	0,91
**Denial of Victim**	0,85	0,92	0,88
**Denial of Responsibility**	0,67	1,00	0,80
**Condemn of the Condemners**	1,00	0,25	0,40
**Appeal to Higher Loyalties**	0,70	0,88	0,78
**Absence of ToN**	1,00	1,00	1,00

### ToN reformulation analysis (Test 3)

The reformulation analysis (Tables 8–10) reveals the model’s high success in rephrasing sentences containing ToN. The model achieved 100% success in reformulating “Appeal to Higher Loyalties” and “Condemning the Condemners” in both Published and Crafted ToN sentences. However, it struggled more with “Denial of Injury”, in both ToN categories.

**Table 8:** Reformulation Analysis for Published Techniques of Neutralization (ToN) Identified (Test 3).

Class	Total	No ToN	ToN left	% Success	Credibility Mean	Credibility SD
**Appeal to higher loyalties**	7	7	0	100	4	0.7559
**Condemning the condemners**	4	4	0	100	3.25	0.433
**Denial of injury**	12	9	3	75	3.7778	0.6667
**Denial of responsibility**	11	9	2	81.8	4.4444	0.7265
**Denial of victim**	12	10	2	83.3	4.2	0.6325

The table presents the total number of sentences for each category of ToN, the number of sentences successfully reformulated without ToN (No ToN), the number of sentences left with ToN after reformulation (ToN left), the success rate of reformulation (% Success), and the mean and standard deviation of the credibility of the reformulated sentences.

**Table 9:** Reformulation Analysis for Crafted Techniques of Neutralization (ToN) Identified (Test 3).

Class	Total	No ToN	ToN left	% Success	Credibility Mean	Credibility SD
**Appeal to higher loyalties**	8	8	0	100	4.375	0.744
**Condemning the condemners**	8	8	0	100	3.875	0.991
**Denial of injury**	12	9	3	75	3.8889	1.6159
**Denial of responsibility**	10	8	2	80	4.75	0.7071
**Denial of victim**	12	11	1	91.7	4.6	0.5045

The table presents the same metrics as Table 8, but for the Crafted ToN sentences.

**Table 10:** Reformulation Analysis for All Techniques of Neutralization (ToN) Identified (Published +  Crafted) (Test 3).

Class	Total	No ToN	ToN left	% Success	Credibility Mean	Credibility SD
**Appeal to higher loyalties**	15	15	0	100	4.2	0.7746
**Condemning the condemners**	12	12	0	100	3.6667	0.8876
**Denial of injury**	24	18	6	75	3.9167	1.1389
**Denial of responsibility**	21	17	4	81	4.5238	0.7496
**Denial of victim**	24	21	3	87.5	4.4167	0.5836

The table presents the same metrics as Table 7 and 8 , but for all ToN sentences combined (both Published and Crafted). Legend of Tables 7–9: Total: Total number of sentences in each category; No ToN: Number of sentences successfully reformulated to remove ToN; ToN left: Number of sentences still containing ToN after reformulation;% Success: Percentage of sentences successfully reformulated; Credibility Mean: Average credibility score of reformulated sentences (1-5 scale); Credibility SD: Standard deviation of the credibility scores.

**Fig 2:**
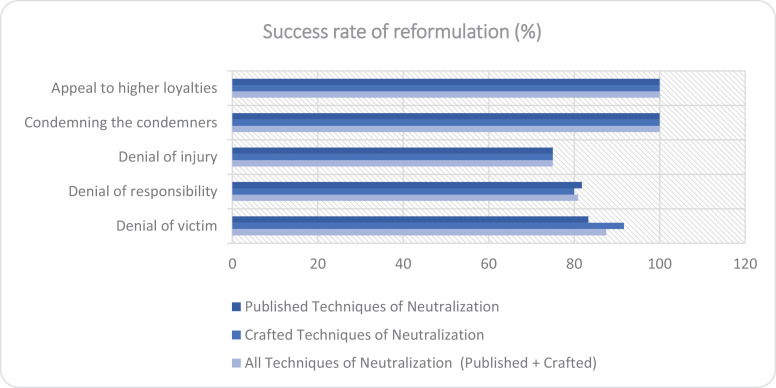
Bar Graph of Reformulation Success Rate for Each Category of Techniques of Neutralization (ToN). This graph visually represents the percentage of successful reformulations for each category of ToN identified in the study (Published, Crafted, All).

**Fig 3:**
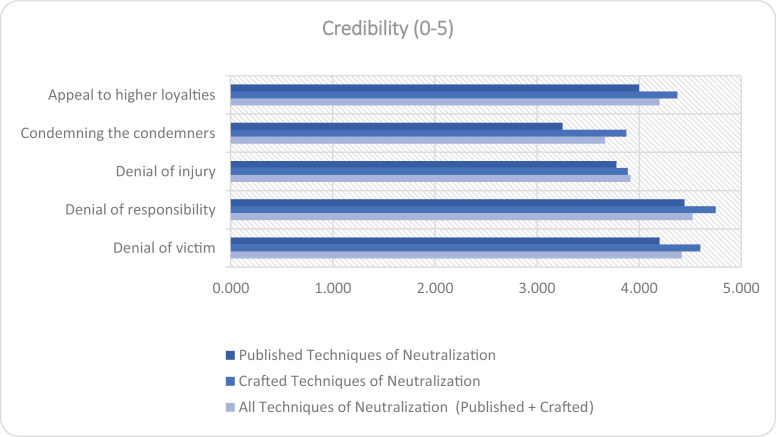
Bar Graph of Credibility of Reformulated Sentences for Each Category of Techniques of Neutralization (ToN). This graph visually represents the credibility scores of the reformulated sentences for each category of ToN identified in the study (Published, Crafted, All).

For a more intuitive understanding of the data, the success rate of reformulation and the credibility of the reformulated sentences are graphically represented in Figs 2 and 3.

## Discussion

The results obtained from the three tests provide valuable insights into the performance of the ChatGPT model in recognizing and reformulating Techniques of Neutralization (ToN). The model demonstrated a high level of accuracy in general ToN recognition, both in Published and Crafted ToN sentences. This is highlighted by the high precision, recall, and F1 scores obtained in both Test 1 (precision of 0.82 and recall of 0.75 for “Denial of Injury”, precision of 0.93 and recall of 1 for “Absence of ToN”) and Test 2 (precision of 1.00 and recall of 0.83 for “Denial of Injury”, precision of 1.00 and recall of 1.00 for “Absence of ToN”). The model’s ability to correctly identify sentences without ToN was also remarkably perfect, indicating its effectiveness in distinguishing neutral sentences from those containing ToN.

Interestingly, the model performed better in recognizing Crafted ToN compared to Published ToN. This could be attributed to the fact that the crafted sentences were formulated in a more “academic” or “scholastic” manner, making the ToN more explicit and easier for the model to identify. This suggests that the model may be more attuned to recognizing ToNs that strictly adhere to their formal definitions.

However, when it came to recognizing specific categories of ToN, the model’s performance varied significantly. The model excelled in identifying categories such as “Denial of Injury”, for which it achieved an F1 score of 0.78 in Published ToN, and “Appeal to Higher Loyalties”, for which it achieved an F1 score of 0.78 in Crafted ToN. These results indicate that the model was able to accurately identify these specific categories of ToN.

On the other hand, the model had significant difficulties with the “Condemn of the Condemners” category. In both tests, the model achieved a low score (for Published ToN, Precision was 0 and Recall was 0, while for Crafted ToN, Precision was 1.00 and Recall was 0.25), indicating that it was unable to correctly identify this category of ToN in either Published or Crafted sentences. This suggests that the model had difficulty discerning this specific category of ToN.

A possible explanation for these performance variations among different ToN categories could lie in the formal definitions of the categories themselves. Some ToN categories, such as “Denial of Injury” and “Appeal to Higher Loyalties”, may have more precise and formal definitions, making it easier for the model to recognize them. Another factor to consider is the amount of data on which the model was trained. Some ToN categories may have been less represented in the training data of ChatGPT, probably because they are less generally present among natural language texts throughout the Internet. This could have influenced the model’s ability to correctly recognize these ToN categories.

Supporting this, empirical research on ToN reveals that among the five categories, Denial of the Victim is the most commonly employed by the criminal population (e.g., perpetrators of theft, robbery, sexual offenses, and homicide), accounting for 70% of cases. This is followed by Denial of Responsibility, Appeal to Higher Loyalties, Denial of Injury, and Condemnation of the Condemners [21–27]. Notably, Condemnation of the Condemners is predominantly observed in samples involving priests and monks who have committed sexual offenses [28,29].

These results suggest that an area for improvement could be further refining the definitions of ToN categories and increasing the amount of training data for less represented ToN categories, to improve the model’s performance.

Our research contributes to the extensive body of literature examining ChatGPT, as illustrated in a preliminary review [30], by addressing for the first time the identification and refinement of criminological elements. This adds a novel perspective to existing analysis of ChatGPT’s capabilities and limitations. While the model exhibits proficiency in a diverse array of natural language processing tasks, it frequently falls short when compared to more sophisticated counterparts. For instance, analysis of over 48,000 prompts reveals ChatGPT’s commendable performance, albeit with notable disparities in comparison to state-of-the-art models (SOTA) (e.g., BERT), especially in nuanced areas such as emotion recognition [31].

Nonetheless, this should not diminish its significance, as ChatGPT remains adept at managing a wide spectrum of tasks. Moreover, the innovative approach of HuggingGPT offers a promising avenue for addressing intricate challenges, showcasing considerable potential towards achieving general artificial intelligence [32].

Furthermore, research indicates that ChatGPT excels not only in Spoken Language Understanding tasks but also in its ability to rectify certain Automatic Speech Recognition errors through its advanced reasoning capabilities [33].

However, it is imperative to acknowledge the hurdles faced by ChatGPT, including challenges in paraphrasing and text similarity tasks, particularly in scenarios involving negative examples [34].

In conclusion, our study aligns with previous research, highlighting common challenges despite the novel application field. For instance, the model faces difficulties in accurately discriminating between subcategories, similar to the challenges noted in emotion recognition studies [31], where models like ChatGPT perform well with general emotional tones but struggle with more nuanced, context-dependent cues.

These insights underscore the ongoing requirement for continued research and development endeavors aimed at mitigating existing limitations and augmenting the capabilities of natural language processing models.

### Limits and future directions

This study represents an initial, exploratory investigation into the applicability of the ChatGPT model in the field of criminology. As such, it provides a preliminary assessment of the model’s ability to recognize, reformulate, and evaluate the credibility of Techniques of Neutralization (ToN) in text. However, it is important to note that this is a starting point, and more robust evaluations are necessary for a comprehensive understanding of the model’s capabilities and limitations in this context.

Future research should include double-blind studies comparing the performance of human evaluators and the AI model in recognizing, reformulating, and assessing the credibility of ToN. This would provide a more rigorous assessment of the model’s performance and could highlight areas where the model excels or falls short compared to human evaluators.

Furthermore, several limitations of the current study should be considered in future research. For instance, the sample used in this study was not uniform, with some ToN categories being less represented than others. This could have influenced the model’s performance and should be addressed in future studies by ensuring a more balanced representation of ToN categories.

Additionally, the study lacked a formal measure of the credibility of the reformulated sentences. Future research should aim to develop and incorporate such a measure to provide a more objective assessment of the model’s performance in this area. Another possible development could involve the integration of more sophisticated word embedding tools to enhance the semantic and quantitative understanding of the text, improve the detection of subtle linguistic patterns, and enable a more nuanced analysis of Techniques of Neutralization.

Another point to consider is the composition of Sample 3 (“Neutral Control Sentences”). In future studies, it would be important to formulate these sentences with varying degrees of “insidiousness” to evaluate the model’s performance under conditions of greater ambiguity.

As a general consideration, during this study, we observed the notably rapid pace at which these systems evolve and spread. This is significant given that scientific inquiry, from idea to publication, inherently involves lengthy processes. We acknowledge that both the experimental protocol and analyses could have been more extensive. However, there is a prevailing sense that, for this particular area of research, time is an essential factor in determining the quality and usability of the work.

## Conclusions

The capabilities of ChatGPT demonstrated in this study point toward several promising potential applications, particularly in the realms of criminology and social psychology. Sykes and Matza [14] first proposed the concept of Techniques of Neutralization (ToN), suggesting that these techniques enable individuals to engage in deviant behavior while minimizing guilt and self-censure. They believed understanding these techniques could offer valuable insights into the criminodynamic processes behind criminal behavior. Utilizing Chat GPT’s proven ability to detect and recast Techniques of Neutralization (ToN), professionals in related fields could enhance their understanding and analysis of written or spoken communication for the presence of such techniques. This could have significant applications not only in forensic contexts, where communication analysis is crucial for investigations but also in the educational sphere [30]. Future criminologists could leverage this tool as part of their training, enabling them to identify and understand ToN more effectively, which is a critical aspect of their professional development.

Furthermore, this technology could be used in preventive and rehabilitative contexts, such as educational programs aimed at reducing recidivism by teaching individuals to recognize and modify maladaptive patterns of which ToN are an expression. In this sense, ChatGPT could be used as an educational tool, helping users understand ToN and functionally reformulate their thoughts. It must be considered that in both situations in which the offender wants to appear rehabilitated or in good faith wants to make use of this type of instrument to work on dysfunctional aspects of their way of thinking, they may find themselves in the condition of only formally ‘overcoming’ the linguistic filter of the use of ToN without really changing their thoughts about what they committed.

While considering these potential applications, it’s also crucial to weigh the potential negative consequences. As with any potent tool, the misuse of ChatGPT can pose risks, as extensively documented in the literature [35–37], particularly in contexts involving criminal actors [38–40]. For instance, Pandey and Patel (2024) explore how ChatGPT could be leveraged by law enforcement but also emphasize its potential misuse in facilitating illegal activities through sophisticated planning and coordination [38]. Similarly, Alawida et al. (2024) highlight the role of ChatGPT in enabling cyberattacks, such as crafting phishing emails or automating malicious code generation, underlining the importance of raising user awareness to mitigate these threats [39]. Esmailzadeh (2023) takes a broader perspective, addressing the implications of ChatGPT misuse in counterterrorism, such as spreading extremist propaganda or simulating fake communications to disrupt security measures [40].

In the specific context of our research, these risks might manifest in scenarios where ChatGPT could assist criminal actors in refining their justifications or even functions as a ‘training tool’ for individuals attempting to mask their usage of Techniques of Neutralization (ToN). This could help them convey their thoughts in a non-minimizing or even empowering way, and manipulate conversations, thereby making the true motivations behind deviant behaviors harder to identify. Additionally, given that these AI models—still in their early stages—exhibit such a significant capability in recognizing and manipulating language, we must stay alert. There’s a risk they could be exploited in new forms of criminal activity or to advance existing ones. This emphasizes the importance of responsible and ethical applications of AI technologies, with an acute awareness of the potential repercussions in the sphere of criminal behavior. Vigilance over the use of generative AI is essential to ensure that these powerful technologies are used responsibly, helping to improve criminological assessment and crime prevention, rather than facilitating the concealment and manipulation of deviant behavior [31]. It is therefore crucial that access to and use of such technologies is carefully regulated and guided by strong ethical considerations and protective measures.

Our test is only one of many possible applications of this AI model and serves mainly as an example to assess its potential and related pitfalls. Overall, even in a field as sensitive and ‘human’ as criminology, AI did not disappoint us. While this study demonstrates the potential of a model like ChatGPT in contributing to the field of criminology, it also highlights the need for a responsible and ethically driven application of AI technologies. As we continue to explore the capabilities of these models, it is necessary to foster the maintenance of a careful balance between benefits and risks, ensuring that they are used in a way that benefits society without compromising individual rights or public safety.

## Supporting information

S1 Data Prompts employed for the task. (DOCX)

S2 Data List of ToN employed in the tests. (XLSX)
