# Supplementary material for: Generative AI and criminology: A threat or a promise? Exploring the potential and pitfalls in the identification of Techniques of Neutralization (ToN)
Source: PLoS One. 2025 Apr 4;20(4):e0319793. doi: 10.1371/journal.pone.0319793 (PMC11970695; doi:10.1371/journal.pone.0319793)
Supplement: S1 Data — (DOCX) [file pone.0319793.s001.docx]

**Prompt**

For the following sentence:


*[SENTENCE]*perform the following steps:

1. **Detection:** Determine whether the sentence contains a Technique of Neutralization (ToN) as defined by **Sykes and Matza (1957).** Answer with "Yes" or "No."
2. **Identification:** If the sentence contains a ToN, identify the specific type of ToN (according to Sykes and Matza, 1957) and provide a brief explanation for your assessment.
3. **Reformulation:** If the sentence contains a ToN, rewrite it to eliminate the ToN while preserving the original meaning as much as possible. If the sentence does not contain a ToN, state: "No reformulation needed."

**Example 1**

**Prompt:**

For the following sentence:

*Now that I’m sober, I’m the most relaxed bloke in the world, my friends would confirm it, but when I’m high on amphetamines, I have poor impulse control, yeah? We, me and my lady, we were high, hadn’t slept for four days. I went to the bathroom, she came in after me and started nagging, you know, and I just couldn’t take it, not just then, I grabbed her arm, hard like, which scared her, I guess, and she hit me”*

perform the following steps:

1. Detection: Determine whether the sentence contains a Technique of Neutralization (ToN) as defined by Sykes and Matza (1957). Answer with "Yes" or "No."

2. Identification: If the sentence contains a ToN, identify the specific type of ToN (according to Sykes and Matza, 1957) and provide a brief explanation for your assessment.

3. Reformulation: If the sentence contains a ToN, rewrite it to eliminate the ToN while preserving the original meaning as much as possible. If the sentence does not contain a ToN, state: "No reformulation needed."

**Answer:**

1 **Detection:**
Yes.

2 **Identification:**
The sentence contains the Technique of Neutralization (ToN) "Denial of Responsibility" as defined by Sykes and Matza (1957).

- **Denial of Responsibility:** The speaker attributes their actions to external factors such as being under the influence of amphetamines and lack of sleep, implying that these circumstances diminished their control over their behavior ("when I’m high on amphetamines, I have poor impulse control" and "we were high, hadn’t slept for four days").

3 **Reformulation:**
"I grabbed her arm during an argument, which scared her, and she hit me. I understand my behavior was my responsibility, regardless of the circumstances."

**Example 2**

**Prompt:**

For the following sentence:

*They expect a bit of banter – doesn't hurt any – just a bit of flirting and a slap on the rump! Nothing serious like.*

perform the following steps:

1. Detection: Determine whether the sentence contains a Technique of Neutralization (ToN) as defined by Sykes and Matza (1957). Answer with "Yes" or "No."

2. Identification: If the sentence contains a ToN, identify the specific type of ToN (according to Sykes and Matza, 1957) and provide a brief explanation for your assessment.

3. Reformulation: If the sentence contains a ToN, rewrite it to eliminate the ToN while preserving the original meaning as much as possible. If the sentence does not contain a ToN, state: "No reformulation needed."

**Answer:**

**1 Detection:**Yes.

**2 Identification:**The sentence contains the Technique of Neutralization (ToN) "Denial of Injury" as defined by Sykes and Matza (1957).

- **Denial of Injury**: The speaker minimizes the impact of their actions by framing them as harmless or insignificant ("doesn't hurt any – just a bit of flirting and a slap on the rump"). This suggests that the actions are trivial and do not cause real harm.

**3 Reformulation:**"I understand that certain behaviors, like flirting or physical gestures, can be perceived as inappropriate or offensive, and I take responsibility for ensuring my actions respect others' boundaries."

**Example 3**

**Prompt**:

For the following sentence:

*I attended a virtual book club and discussed a thought-provoking novel.*

perform the following steps:

1. Detection: Determine whether the sentence contains a Technique of Neutralization (ToN) as defined by Sykes and Matza (1957). Answer with "Yes" or "No."

2. Identification: If the sentence contains a ToN, identify the specific type of ToN (according to Sykes and Matza, 1957) and provide a brief explanation for your assessment.

3. Reformulation: If the sentence contains a ToN, rewrite it to eliminate the ToN while preserving the original meaning as much as possible. If the sentence does not contain a ToN, state: "No reformulation needed."

**Answer:**

1. **Detection:**
   No.
2. **Identification:**
   The sentence does not contain a Technique of Neutralization (ToN) as defined by Sykes and Matza (1957).
3. **Reformulation:**
   No reformulation needed.
